# Double-edged sword: The evolutionary consequences of the epigenetic silencing of transposable elements

**DOI:** 10.1371/journal.pgen.1008872

**Published:** 2020-07-16

**Authors:** Jae Young Choi, Yuh Chwen G. Lee

**Affiliations:** 1 Center for Genomics and Systems Biology, Department of Biology, New York University, New York, New York State, United States of America; 2 Department of Ecology and Evolutionary Biology, University of California, Irvine, California, United States of America; University of Liverpool, UNITED KINGDOM

## Abstract

Transposable elements (TEs) are genomic parasites that selfishly replicate at the expense of host fitness. Fifty years of evolutionary studies of TEs have concentrated on the deleterious genetic effects of TEs, such as their effects on disrupting genes and regulatory sequences. However, a flurry of recent work suggests that there is another important source of TEs’ harmful effects—epigenetic silencing. Host genomes typically silence TEs by the deposition of repressive epigenetic marks. While this silencing reduces the selfish replication of TEs and should benefit hosts, a picture is emerging that the epigenetic silencing of TEs triggers inadvertent spreading of repressive marks to otherwise expressed neighboring genes, ultimately jeopardizing host fitness. In this Review, we provide a long-overdue overview of the recent genome-wide evidence for the presence and prevalence of TEs’ epigenetic effects, highlighting both the similarities and differences across mammals, insects, and plants. We lay out the current understanding of the functional and fitness consequences of TEs’ epigenetic effects, and propose possible influences of such effects on the evolution of both hosts and TEs themselves. These unique evolutionary consequences indicate that TEs’ epigenetic effect is not only a crucial component of TE biology but could also be a significant contributor to genome function and evolution.

Transposable elements (TEs) are selfish genetic elements that can copy themselves and move to another genome location. Because of TEs’ replicative nature and mobile behavior, they are prevalent across eukaryotic genomes in both the transcriptionally silenced heterochromatin and the gene-rich euchromatin [1]. This distribution of TEs, not surprisingly, could have significant functional and fitness consequences, such as disrupting functional sequences [2–4] and providing ectopic regulatory elements [5,6]. Ectopic recombination between nonallelic TE insertions also leads to highly detrimental chromosomal rearrangement [7–9]. A common thread among these well-studied deleterious consequences of TEs is that they are mediated by TEs’ “genetic” effects (e.g., disruption of DNA or changes of DNA sequences). On the other hand, recent evidence suggests that TEs can also have potent “epigenetic” effects on host function and fitness. The term “epigenetics” has a wide variety of meanings [10], and we use a more specific definition: changes in the heritable states of chromatin that are independent of the underlying DNA sequences [11]. In this Review, we summarize the recent advances in understanding the long-overlooked epigenetic effects of TEs in natural populations and how these effects have shaped the evolution of both TEs and host genomes.

## Epigenetic effects of TEs: Cause

In eukaryotes, genomic DNA and histones complex into chromatin, which can further fold and compact into higher-order structures inside the cell nuclei. Biochemical modifications of DNA or histones leave molecular markers that encode “instructions” for gene regulation [12]. Based on the distribution of these epigenetic modifications, the eukaryotic genome is grossly categorized into gene-rich, transcriptionally active euchromatin and gene-poor, transcriptionally silent heterochromatin. In organisms with monocentric chromosomes, constitutive heterochromatin is predominantly located around centromeres (pericentromeric) and at the ends of chromosomes (peritelomeric) and is enriched with repressive epigenetic marks, such as DNA methylation and specific modifications of histone tails, in particular, di- and tri-methylation on lysine 9 of histone H3 (H3K9me2/3) [13–15] (but see [16,17] for the heterochromatin of “holocentric chromosomes,” which has similar but also many different properties from the heterochromatin of monocentric chromosomes). These repressive epigenetic marks promote the packaging of chromatin into compacted nuclear compartments. In addition, these heterochromatic compartments can phase separate from the euchromatic genome, making constitutive heterochromatin inaccessible to various cellular processes, such as transcription, replication, and DNA damage repair (reviewed in [18]). Outside the transcriptionally silenced heterochromatin, the repressive epigenetic marks are also found in the gene-rich and transcriptionally active euchromatin [19–22], particularly around euchromatic TE sequences.

**Fig 1:**
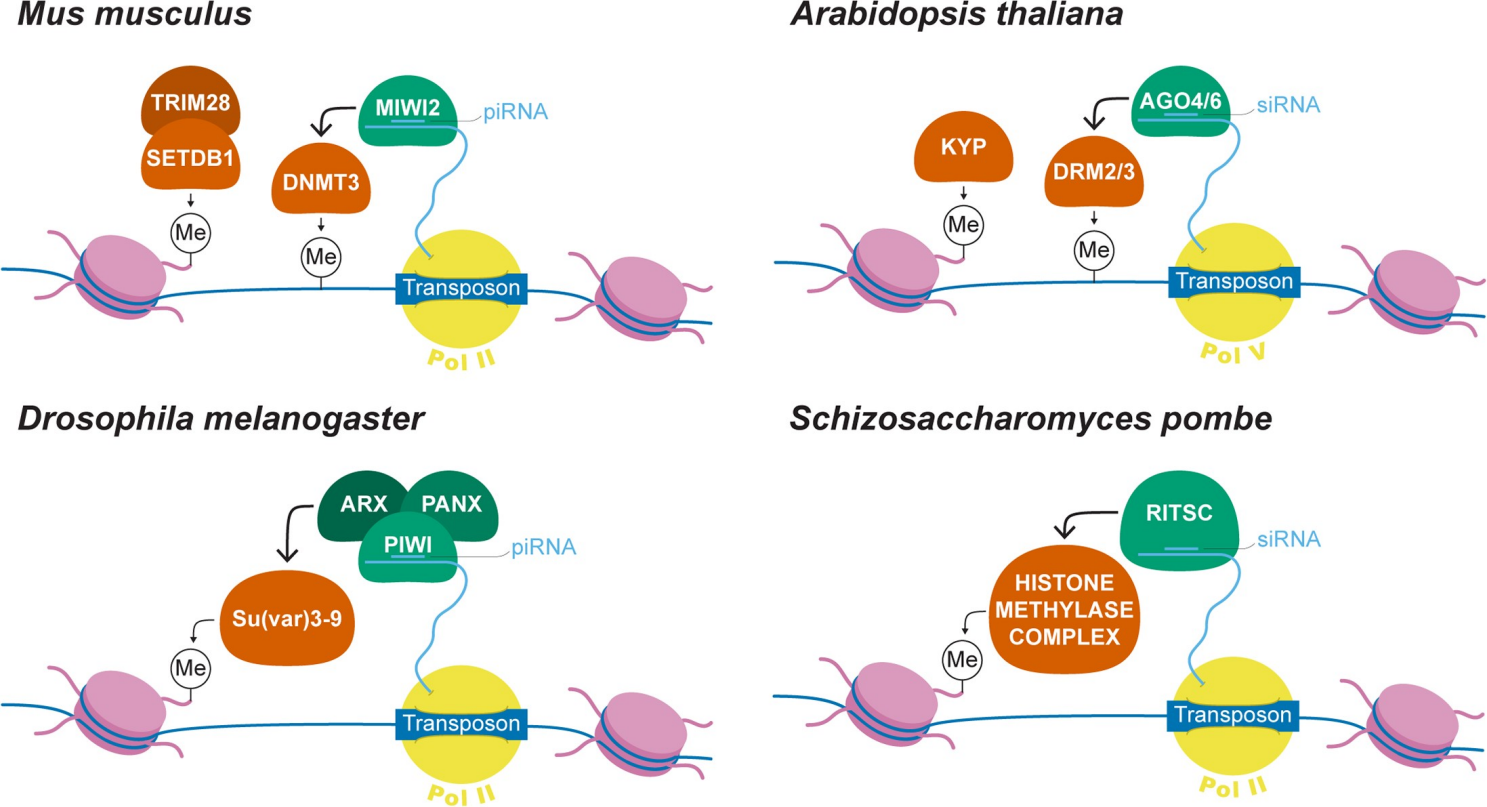
Mechanisms for the epigenetic silencing of TEs in animals and plants. To portray the similarities of the mechanism underlying TE epigenetic silencing between species, this figure only shows core proteins involved in the processes for four representative eukaryotic species. Detailed mechanisms and involved proteins could be found at other detailed reviews (e.g., [134,134–136]). In mammals, piRNAs are loaded onto MIWI2, which promotes a chromosomal environment that ultimately recruits the mammalian DNMT3 to methylate TE sequences [137,138]. Complementarily, KAP1, also called TRIM28, recognizes retroviral TE sequences by binding to a conserved primer binding site [139] and recruits H3K9 methyltransferase SETDB1 and HP1 to form a repressive heterochromatic environment at TE sequences [140–142]. Drosophila lacks DNA methylation, but TEs can still be epigenetically silenced through histone modifications. Nascent TE transcripts are targeted by piRNA loaded PIWI, which complexes with Asterix (Arx, also known as Gtsf1) and Panoramix (Panx, also called Silencio) [143–146]. This complex recruits Su(var)3-9, a key H3K9 methyltransferase, to lay down repressive H3K9me2/3 at TE sequences [147]. A similar mechanism is also observed in Schizosaccharomyces pombe, where RITSC binds to nascent TE transcripts through base-pairing with siRNAs and recruits histone methyltransferase complex to methylate H3K9 histone tails [148]. In plants, euchromatic TEs are silenced through the RdDM pathway [168]. AGO4 or AGO6 are guided by siRNA [149,150] and targeted to siRNA-matching regions through scaffolding RNA that were transcribed by Pol V [151–153]. This double-stranded RNA further recruits methyltransferases DRM1 and DRM2 (homolog to the mammalian methyltransferase DNMT3 [154]), resulting in methylation of TE sequences [155,156]. AGO4, argonaute 4; DNMT3, DNA methyltransferase 3; DRM,1, domains rearranged methylase 1; KAP1,; KRAB-associated protein 1 KRAB, Krüppel-associated box; MIWI2, mouse piwi 2; pRNA, PWI-interacting RNA; RdDM, siRNA-directed DNA methylation; RITSC, RNA-induced transcriptional silencing complex; siRNA, small interfering RNA; TE, transposable element.

**Fig 2:**
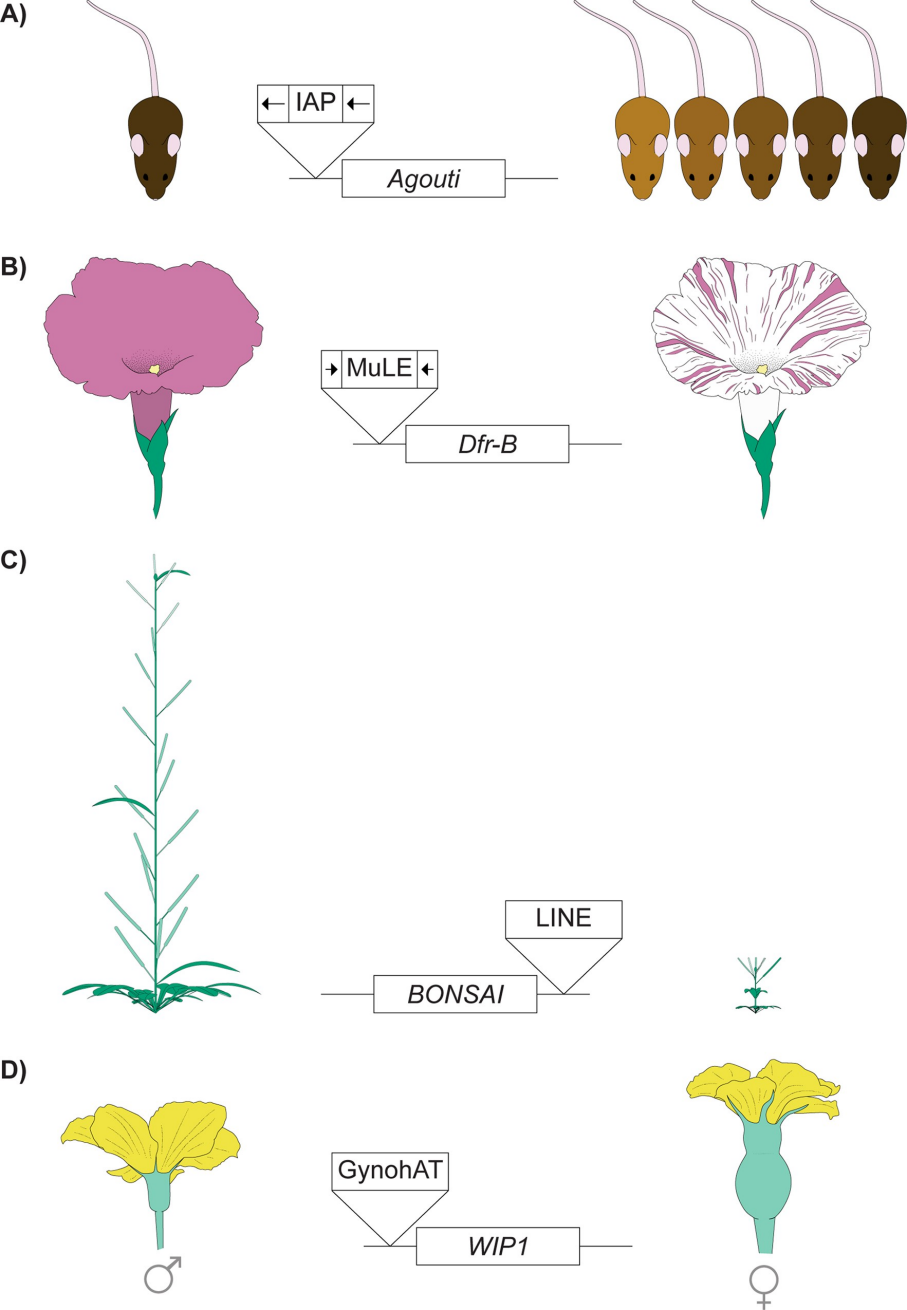
Phenotypic variation hints TEs’ epigenetic effects. (A) In mice, an IAP retrotransposon inserted upstream of agouti locus results in ectopic transcription of the gene that was initiated within the IAP, leading to yellow fur [157]. Curiously, isogenic mice with this IAP insertion were observed to display a wide spectrum of coat color, from yellow, mixture of yellow and agouti, to agouti, which was attributed to the varying epigenetic states of the IAP element [24]. Similar observations are also abundant in plants. (B) Variegated methylation of the promoter of Dfr-B, a flower color gene, results in color streaks in morning glory, and this is associated with the presence of a nearby MuLE DNA element [25]. (C) A LINE element in the 3’ noncoding regions causes the hypermethylation of bonsai gene, which silencing the gene and leading to bonsai-like A. thaliana [26]. (D) In muskmelon (Cucumis melo), the sex determination of flowers is associated with an insertion of GynohAT DNA element in the proximity to WIP1, which encodes a transcription factor that promotes male flower development. The presence of the TE leads to hypermethylation of WIP1 and thus female flowers [27]. IAP, intracisternal A-particle; LINE, long interspersed nuclear elements; TE, transposable element.

To safeguard the genomes from the harmful effects of TEs, both animals and plants can epigenetically silence TEs through small-RNA–mediated mechanisms (**Fig 1**) (reviewed in [23]). Specifically, PIWI-interacting RNAs (piRNAs) or small interfering RNAs (siRNAs) guide the RNAi protein complex to euchromatic TEs with complementary sequences. The complex then recruits DNA and/or histone methyltransferases to modify the DNA or histone tails associated with the TE sequences, resulting in an enrichment of repressive epigenetic marks at TEs (**Fig 1**). This epigenetic silencing of TEs reduces the selfish increase of TEs, ultimately benefiting the host. However, several early studies on the genetic basis of a wide array of phenotypic traits hinted at how the epigenetic silencing of TEs could, in turn, influence nearby host genes (**Fig 2,** [24–27]). A common feature of the observations in **Fig 2** is that the presence of a TE insertion, irrespective of TE type, was associated with the silencing of nearby genes. A reporter transgene study in *Drosophila melanogaster* provides a clue for the potential mechanism underlying this phenomenon [28]. It was observed that the strength of silencing a randomly inserted transgene in the euchromatic genome inversely correlates with the distance between the transgene and a DNA-based TE family named *1360*. This observation is consistent with the predictions of the “mass action model” [29], which has been applied to explain the distance-dependent spreading of repressive epigenetic marks from pericentromeric and peritelomeric heterochromatin [13,30,31]. This model proposed that the extent of heterochromatin assembly depends on the concentration of heterochromatic structural and enzymatic proteins, whose destiny is expected to be the highest at heterochromatin and decreases with distance. Epigenetically silenced TEs are likely another kind of heterochromatin-nucleation center and could similarly result in distance-dependent enrichment of repressive epigenetic marks around TEs. Another nonexclusive mechanism that may mediate the observed spreading effects of TEs is the generation of small RNAs that target both TEs and neighboring sequences. Indeed, in *Drosophila*, euchromatic TEs become de novo piRNA generating loci, and the generation of small RNAs can extend into TE-flanking regions [32]. In plants, targeting of small RNAs can fuel the production of additional small RNAs from the neighboring sequences, a phenomenon known as “transitivity” [33,34]. In both scenarios, the probability of TE-flanking sequences being targeted by the epigenetic silencing pathway dwindles with the increased distance to TE.

Recent waves of epigenomic studies further support the idea that the spreading of repressive marks from euchromatic TEs is not an isolated event and is surprisingly prevalent within genomes and across taxa. A study in *Arabidopsis thaliana* first demonstrated that 500 bp windows flanking TEs are enriched with suppressive DNA methylation [35]. Similarly, in mice, it was observed that there is not only an enrichment of H3K9me2 around TEs, but the enrichment level decreases with increased distance from TEs, demonstrating a striking distance-dependent effect [36]. Up to now, genome-wide evidence for TE-associated spreading of repressive marks has been documented in a wide variety of species, including *A*. *thaliana* [37,38], rice [39], maize [40–43], *Drosophila* [44–46], and mice [36]. In specific cases, this spreading of repressive marks can extend beyond 20 kb from euchromatic TE insertions and is detected in a large proportion of euchromatic TEs (e.g., more than 50% in *Drosophila*) [46]. Several of these studies characterized the epigenetic landscapes around polymorphic TEs in multiple individuals of the same species [36–38,40,46], which allowed the comparison of epigenetic states of homologous sequences in the presence and absence of TEs. These analyses support that the enrichment of repressive marks around TEs are mediated by TEs themselves, instead of resulting from TEs’ preferential insertions into genomic regions that are already enriched with repressive marks (but see [43] for an exception in maize).

Together, these taxonomically diverse studies reveal similarities but also interesting differences in TE-mediated spreading of repressive marks. Within species, the level of spreading varies greatly between TE families, with long terminal repeat (LTR) families showing the most dominant spreading effects in multiple taxa [36,43,46]. Because of the involvement of small RNAs in the epigenetic silencing of TEs, it is not surprising that there is a positive association between small RNAs targeting and the extent of TE-mediated spreading effects in both plants [47] and flies [45,46]. However, even for TE families that overall show the strongest spreading effects, some of their insertions have limited spreading of repressive marks [36,43,46]. Interestingly, the average extent of spreading varies between species, with an average of a hundred base pairs in *A*. *thaliana* [37,38], about 1 kb in maize [40,42], and up to 4–5 kb in mouse and *Drosophila* [36,45,46]. While it is possible that differences in experimental techniques and analysis methods might have contributed to some of the observed between-taxa differences, these findings raise the important question of what evolutionary processes underlie the variability of TE-mediated spreading of repressive marks within and between species, which we discuss below.

### Epigenetic effects of TEs: Known consequences to date

Can the spreading of repressive marks from TEs (i.e., “TEs’ epigenetic effects”) extend into functional sequences and influence host fitness? In *Drosophila*, TEs with spreading effects are associated with higher enrichment of H3K9me2 at their neighboring genes when compared to homologous alleles without nearby TEs [46]. Similarly, in *A*. *thaliana* wild populations, TEs are major associates of differentially methylated regions (DMRs), many of which cover genes [37,38,48–50]. These observations support the notion that the spreading of repressive marks from TEs can extend into nearby functional elements. Analysis of the genome-wide distribution and population frequencies of TEs further reinforces the possibility that TEs’ epigenetic effects can reduce host fitness and consequently must have functional impacts. If the spreading of repressive marks from TEs into genes is deleterious, there should be a dearth of silenced TEs near genes due to purifying selection removing these TEs. Consistent with this prediction, across several plant species, methylated TEs are more distant from genes than unmethylated TEs [39,40,51,52]. Another approach to infer the fitness impacts of TEs is through analyzing TEs’ population frequencies, which negatively correlate with the strength of natural selection removing TEs from the population [53–55]. Compared to other TEs, low-frequency TEs, which should be more strongly selected against, are more likely to be methylated in *A*. *thaliana* [51]. Similarly, in *D*. *melanogaster*, low-frequency TEs are associated with more extensive spreading of H3K9me2 [46] as well as higher enrichment of H3K9me3 at neighboring genes across multiple developmental stages [45]. Importantly, these studies not only provide support for the deleterious fitness consequences of TEs’ epigenetic effects but also paint a picture where the magnitude of impact on the host fitness is determined by the extent and strength of TE-mediated spreading of repressive marks.

The most obvious functional consequences of TEs’ epigenetic effects that could impact host fitness is the perturbation of neighboring gene expression. Genetic manipulations that disrupted the epigenetic silencing of TEs result in a lowered enrichment of repressive epigenetic marks and increased expression of TE-neighboring genes in *Drosophila* [44,56], indicating that TE-mediated spreading of repressive marks reduces the expression of neighboring genes. Studies using individuals derived from natural populations suggest a similar picture. A pioneering study in *A*. *thaliana* first reported negative correlations between the methylation density of TEs and the expression of their adjacent genes [51]. A follow-up study further found that silenced TEs are associated with divergence in gene expression between *A*. *thaliana* and the closely related *A*. *lyrata* [57]. Associations between silenced TEs and lower expression at adjacent genes were also reported within the genomes of maize [40], flies [45], and rice [39]. These single-genome analyses support an overall dampening effect of TE-mediated spreading of repressive marks on gene expression. However, studies that jointly analyzed mobilomes, epigenomes, and transcriptomes of multiple individuals from the same species suggest a more convoluted relationship between TEs’ epigenetic effects and the expression of neighboring genes. In *A*. *thaliana*, comparisons between homologous alleles with and without TEs found that the methylation status of TEs results in not only the expected decreased but also quite frequently increased expression of TE-adjacent genes [37,38]. Similarly, associations between gene expression levels and the extent of TE-mediated spreading of H3K9me2 in a *Drosophila* study were weak [46]. These observations could result from the complex role of epigenetic modifications in gene regulation. For instance, large-scale population epigenomic studies in *A*. *thaliana* reported a limited effect of naturally varying levels of DNA methylation on gene expression [50,58]. The sensitivity of transcription to repressive epigenetic marks also varies between genes. In *Drosophila*, genes within a local euchromatic neighborhood respond very differently to the ectopic enrichment of heterochromatic marks, with only some genes showing strongly reduced expression [59]. In fact, the enrichment of key heterochromatic proteins, paradoxically, has been shown to be necessary for the proper transcription of some genes located in not only the pericentromeric heterochromatin [60–62] but also the euchromatic genome [63,64]. Still, the transcriptional consequences of TEs’ epigenetic effects could depend on other factors that have yet to be jointly studied, such as insulator sequences, other histone modifications, or other types of variants (single nucleotide polymorphisms (SNPs) or copy-number variants). It is worth noting that TEs with epigenetic effects that strongly silence their neighboring genes are unlikely to be sampled because selection against their harmful effects would have resulted in their low frequencies in natural populations. Even if sampled, selection against the recessive deleterious effects of these TEs could also lead to their removal during the establishment of inbred strains or homozygous accessions in the lab. The transcriptional effects of TE-mediated silencing that are left for investigators would thus be much weaker and require more sensitive assays.

Alternatively, other *genetic* effects of TEs could also contribute to perturbed gene expression. For instance, in *D*. *melanogaster*, random hopping of foreign TE families (i.e., TE families that are not naturally present in the focal species) successfully recovered many gene expression mutants in which TEs are inserted at a distance from the impacted genes [65,66]. These foreign TE families are unlikely targeted by the small-RNA–mediated silencing (e.g., [67]), and their transcriptional influence should have been mediated by mechanisms other than the spreading of repressive marks from TEs, such as the disruptions of regulatory sequences. TEs also contain various regulatory elements (e.g., enhancers and insulators) that can disrupt or enhance neighboring gene expression (reviewed in [5]). In addition, epigenetically silenced TEs are recently found to change the higher-order structure of genomes, which also has the potential to influence the transcription of TE-adjacent genes [68]. The same study, however, found that most TEs that changed the 3D genome structure also lead to the spreading of repressive marks. These transcriptional effects of TEs and TE-mediated spreading of repressive marks all act in cis to TE-neighboring genes, making it challenging to disentangle their effects on gene expression. Nevertheless, evolutionary studies still reveal that TEs with epigenetic effects are more strongly selected against than TEs without the effects (see above), supporting the functional importance of TE-associated spreading effects.

### Selection against the epigenetic effects of TEs influences TE evolution

From the studies highlighted above, it is apparent that TEs’ epigenetic effects impair host fitness. Selection against the deleterious impacts of TEs has been widely demonstrated to play a critical role in the evolutionary dynamics of TEs [53–55]. Based on the molecular mechanisms of epigenetic silencing, we propose that selection against TEs' effects could have several unique influences on TE evolution, which we discuss below.

**Fig 3:**
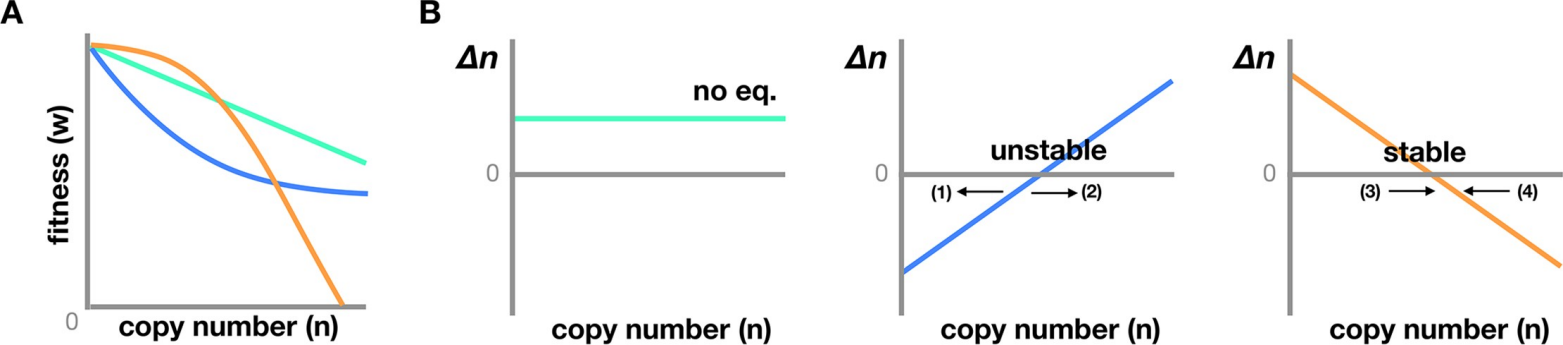
Impacts of the epistatic interactions among TEs’ deleterious effects on TEs’ population dynamics. (A) Three possible models for the interactions among TEs’ deleterious effects: additive (green), multiplicative (blue), and synergistic (orange) models (reviewed in [54]). (B) The per-generation change in TE copy number (Δn) under these models when the transposition rate of TEs holds constant. Theories predict that Δn is determined by the rate of TE replication (increases copy number) and the rate of changes in host fitness (decreases copy number by selection against TEs’ deleterious effects) [69]. Additive model: When the deleterious effects of TEs are independent, host fitness is determined by summing the effects of individual TE in the host genome. In this case, host fitness declines linearly with respect to TE copy number (green in A), and the rate of fitness change with every additional TE holds constant (the slope of green line in A). Unless the rate of fitness change is exactly the same as the rate of increase in TE copy number through transposition, no equilibrium could be reached (B left panel). On the contrary, with epistatic interactions among TEs’ deleterious effects (multiplicative or synergistic), the relationship between host fitness and TE copy number is nonlinear, and equilibrium in TE copy number is possible. Multiplicative model: The deleterious effects of TEs weaken with each additional TE in a host genome (blue in A), and the rate of fitness change decreases with increased TE copy number (slope of blue line in A). While TE copy number could reach equilibrium (i.e., when Δn = 0, B middle panel), this equilibrium is unstable. A slight decrease in TE copy number leads to an even faster decline in TE copy number (arrow [1]) while a slight increase has the opposite effects (arrow [2], B middle panel). Synergistic model: Each additional TE imposes a larger fitness cost than the last TE, and the rate of fitness change escalates with more TEs in a host genome (slope of orange line in A), which accelerates the removal of TEs from the population. A stable equilibrium is possible because slight perturbations always lead back to the equilibrium (arrow [3] and [4], B right panel). TE, transposable element.

A central question in the evolutionary dynamics of TEs is how TEs are contained in host populations over evolutionary time. Selection against TEs’ epigenetic effects may play a previously underappreciated role in ensuring a stable containment of TEs. Classical theory predicts that reducing the transposition rate to equal the rate of TE removal alone is not sufficient to have a stable equilibrium of TE copy number [69,70]. Slight changes in the rate of TE replication or removal could lead to drastic changes in TE copy number (**Box 1** and **Fig 3**). On the other hand, when the deleterious effects of TE insertions are interdependent and each additional TE exacerbates host fitness with a larger effect than the last TE, or synergistic epistasis of TEs’ fitness effects (**Box 1** and **Fig 3**), a stable equilibrium of TE copy number is possible [69]. While classic mutation accumulation experiments ([71], reviewed in [72]) and recent population genetic studies [73] support synergistic epistatic effects of deleterious mutations, whether the fitness effects of TE insertions conform to those of simple mutations is still an open question. In fact, only ectopic recombination between TEs (one of the many genetic effects of TEs) has been indirectly supported to have synergistic fitness effects [7,74]. Nevertheless, the dependency of TE silencing on small RNAs and the key role of TE transcripts in the generation of small RNAs have invited the predictions that the deleterious epigenetic effects of TEs potentially provide the required synergism for the stable containment of TE copy number (**Box 1**).

**Box 1. Synergistic epistasis of the deleterious epigenetic effects of Tes**

General models for the evolutionary dynamics of TEs predict that the copy number of a TE family is determined by processes that increase (transposition) and decrease (excision and selection) TE copy number in an outbreeding host population [69,70]. When the transposition and excision rates are independent of TE copy number (*n*), the approximate change in TE copy number per generation (*Δn*) depends on the mode by which host fitness changes with respect to TE copy number. Under the additive model, the fitness impact of all TEs is simple additivity of their individual effect, and a linear model describes the relationship between host fitness and TE copy number (**Fig 3A**). Epistatic interactions among the fitness effects of TEs lead to a deviation from this linear relationship, and different modes of the interaction could have distinct impacts on the evolutionary dynamics of TEs (**Fig 3B)**. Specifically, theories have suggested that synergistic epistasis among TEs’ deleterious effects leads to a stable containment of TE copy number (**Fig 3B**, [69]).

The epigenetic silencing of TEs implicates two possible mechanisms by which synergistic epistasis among TEs’ deleterious epigenetic effects could arise.

**Mechanism I**

In both plants and animals, the generation of TE-targeting small RNAs depends on the transcription of TEs. In plants, Dicer-like3 (*DCL3*) processes TE transcripts into 24nt siRNAs [158,159]. In mammals and insects, TE transcripts play an important role in ping-pong cycle for the generation and amplification of piRNAs [160–162]. The amount of small RNAs targeting a TE family should thus depend on the copy number of the very same family, which is supported by empirical studies in *Drosophila* [163]. Consistent with the fact that these small RNAs initiate the epigenetic silencing of TEs, the strength of TEs’ epigenetic effects has been observed to positively correlate with the amount of small RNAs targeting a TE family [45,46].

A simple linear relationship between the amount of small RNAs targeting a specific TE family (*r*) and copy number of that family (*n*) can be expressed as:

r=an, (1)

where *a* captures the rate of small-RNA generation from each TE. Assuming that the amount of small RNAs is in excess and the targeting of a TE by small RNAs will not influence the probability of another TE insertion being targeted, the probability of epigenetically silencing a TE (*P_silenced_*) depends on the amount of small RNAs (*r* in e.q., 1) and, accordingly, increases with the copy number of that TE family. The number of silenced TEs can be described as:

$$n_{silenced}=P_{silenced}n=brn=(ab)n^{2}.$$ (2)

Here, *b* summarizes the effect of a unit of small RNAs having on the epigenetic silencing of TEs. If each epigenetically silenced TEs have probability *c* to result in the spreading of repressive marks, which reduces host fitness by *s*, the host fitness (*w*) can be expressed as:

$$w=1-s(cn_{silenced})=1-(sabc)n^{2}.$$ (4)

Host fitness will decrease faster than linearly with respect to an increase in family copy number. In other words, each additional TE would impose a larger detrimental effect than the last TE, leading to synergistic epistasis among TEs’ deleterious epigenetic effects.

This nonlinear relationship between the number of silenced TEs and copy number of TE family has been hinted in some empirical studies. The strength of epigenetic silencing of a TE family positively correlates with family copy number in plants [164–166]. Similarly, in *Drosophila*, the proportion of TEs with *cis-*spreading of repressive marks also increases with family copy number [46]. Yet, several assumptions of this model still need further empirical investigations (e.g., the amount of small RNAs is in excess). Future experimental analyses will help evaluate these assumptions as well as test the generality of the observed relationship between family copy number and the number of silenced Tes and the link of that to host fitness.

**Mechanism II**

In most animals, the generation and amplification of piRNAs are mediated through the “ping-pong cycle” (reviewed in [167]). Using ping-pong cycle in *Drosophila* as an example, transcripts of euchromatic TEs are targeted and cleaved by *PIWI/AUB*, which are guided by antisense piRNAs, leading to the generation of sense piRNAs. These sense piRNAs are then loaded onto another *Piwi* protein, *AGO3*, which targeted and cleaved the primary transcripts of “piRNA clusters” (TE-dense loci in heterochromatin) to generate even more antisense piRNAs (reviewed in [160–162]). This positive feedback loop of piRNA amplification suggests that the amount of piRNA targeting a TE family may grow quadratically or even exponentially with family copy number and can also lead to the synergism of the deleterious epigenetic effects of TEs. This nonlinear relationship between the amount of piRNA and family copy number has been long predicted [54]. However, alternative scenarios (such as the amplification of piRNAs has a regulated maximum or the amount of primary transcripts of piRNA clusters are limited—and thus limiting an important component of the ping-pong cycle) are also possible and would result in different predicted dynamics. Quantification of the absolute amount of piRNAs in relation to TE copy number and empirical investigations of the dynamics of ping-pong cycle will help further test the predicted nonlinear relationship between the amount of piRNAs and TE copy numbers and the synergistic epistasis arising from TEs’ epigenetic effects.

Selection against the deleterious epigenetic effects of TEs may also be a potent evolutionary force shaping divergent TE profiles between species. A comparison between *Drosophila* species found that *D*. *simulans* TEs have stronger epigenetic effects than TEs in its closely related *D*. *melanogaster* [46]. This is expected to result in stronger selection removing TEs in *D*. *simulans*, which is consistent with the reported lower genomic TE content in the species than in *D*. *melanogaster* [75,76]. A similar negative association between TE abundance and the strength of TEs’ epigenetic effects is also observed within the *Arabidopsis* family. Compared to its sister lineage *A*. *lyrata*, *A*. *thaliana* has a smaller genome that is depauperate of TE sequences [77]. TEs in *A*. *thaliana* are also targeted by more siRNAs and have stronger transcriptional consequences on adjacent genes, both of which suggest more substantial epigenetic effects of TEs [57]. Future investigations that extend to more species across the phylogeny would help test the generality of these observations and evaluate the role of TEs’ epigenetic effects in the evolutionary divergence of genomic TE content.

The nature of purifying selection against TEs’ deleterious epigenetic versus genetic consequences may also differ. Epigenetic silencing varies throughout development and changes depending on environmental conditions [13,78,79]. TEs’ epigenetic effects may also show these variabilities, which is evidenced by TE-induced mosaic phenotypes (**Fig 2**) and the differing extent of TE-associated spreading of repressive marks documented across developmental stages [45] and at different temperatures [49]. The deleterious phenotypes arising from TEs’ epigenetic effects, and selection against these effects, would thus vary depending on context and condition, which is similar to selection against deleterious mutations under fluctuating environment. Theoretically, when the fitness effects of a mutation change over various environmental conditions an organism experiences, selection is less effective at distinguishing alleles that are, on average, deleterious from other mutations [80]. Accordingly, selection may be overall less effective at removing TEs with harmful epigenetic effects when compared to TEs with similar magnitude of genetic effects that are context independent. This is an attribute that needs to be further incorporated into theoretical modeling to define its influence and importance on the evolutionary dynamics of TEs.

### Epigenetic effects of TEs on host genome evolution: Impacts on recombination

TEs were recently proposed as potential modifiers of the recombination landscape [81], which is especially plausible with TE-mediated local enrichment of repressive epigenetic marks. Recombination is strongly reduced at pericentromeric and peritelomeric heterochromatin [82–84]. The enrichment of repressive epigenetic marks and the associated compacted phase-separated chromatin structure (see above) are likely explanations for this observation. The distribution of double-stranded breaks (DSBs), which are generated by *Spo11* and initiate recombination [85,86], is strongly associated with open chromatin across the genomes of both animals [87–89] and plants [90,91]. Increased *Spo11* localization [92] and elevated recombination events [93] at pericentromeric heterochromatin have been observed in *A*. *thaliana* mutants that abolished H3K9me2/3 and/or DNA methylation (but see [94]). Similarly, mutants leading to loss of H3K9me2/3 have an increased number of DSBs in the pericentromeric regions in *Drosophila* oocytes [95] and in fission yeast [96]. These studies indicate that DSB initiation is suppressed in genomic regions enriched with repressive marks. In addition, other steps of the recombination pathways could similarly be influenced by repressive chromatin states, although studies in mitotic cells currently suggest a positive effect of heterochromatin marks on homologous recombination [97,98].

However, the enrichment of heterochromatic marks and the associated recombination suppression at pericentromeric and peritelomeric regions are of Mb scale. With the much smaller footprint of enrichment of repressive epigenetic marks, it is still an open question whether a silenced euchromatic TE is sufficient to suppress recombination and alter the recombination landscape. Some evidence suggests that recombination can be suppressed by the presence of repressive marks at a much smaller scale than that of constitutive heterochromatin. For instance, de novo DNA methylation of a 7.5 kb nonrepetitive region in fungus results in over a hundred-fold reduction in cross-over frequency [99]. In mice, recombination of a transgene targeted by the V(D)J recombinase is also inhibited by ectopic DNA methylation [100]. In addition, it was observed that the recombination rate in an intergenic, nonrepetitive sequence was reduced by the insertion of a TE in maize [101,102], and TEs’ epigenetic effect is a tentative explanation. Other evidence comes from studies that experimentally manipulated the chromatin states of TEs. Loss of DNA methylation at TEs leads to an elevated number of DSBs forming inside TEs in mouse meiotic cells [103]. Increased *Spo11* occupancy at TEs is also observed in *A*. *thaliana* DNA methylation mutants, although this effect is restricted to a subset of TEs [92]. These observations suggest that TE-induced enrichment of repressive epigenetic marks, even at a kb scale, can potentially modify local recombination rates.

Recombination plays a crucial role in genome evolution. It influences the efficacy of both positive and negative selection, significantly shaping genome-wide patterns of genetic variation and adaptive evolution [104]. Within euchromatic genomes, recombination rates fluctuate along chromosomes [105–108] and the epigenetic effects of TEs could be an important contributor to such patterns. It is worth noting that there is a great variability in the insertion positions of TEs not only between species [76,77] but also within populations. In fact, the majority (more than 90%) of the TEs that are inserted at a specific genomic location are not present in all individuals in both animal [109–112] and plant [37,38,113,114] populations. Although generally selected against, deleterious TEs could persist in host populations for an appreciable amount of time (e.g., [115,116]). If the magnitude of TEs' suppressing effects on recombination is at a similar order of magnitude to other evolutionary processes (e.g., selection), the prevalent polymorphic TEs may result in varying recombination landscapes that could profoundly shape genome evolution within and between species, which is a hypothesis worthy of further investigation.

### Evolution of the epigenetic effects of TEs

Host-directed silencing of TEs reduces TEs’ selfish replication, minimizes the harmful impacts of TEs, and ultimately benefits hosts. However, such silencing also triggers inadvertent and harmful spreading of repressive marks from TEs. In other words, TEs are deleterious because of host silencing mechanisms evolved to reduce TEs’ detrimental impacts. Then, why have organisms failed to curb the associated spreading effects?

The spreading of repressive marks from TEs is likely under host control in some systems. In *A*. *thaliana*, demethylation genes (*DML2*, *DML3*, *and ROS1*) actively maintain the boundaries of DNA methylation between TE-induced heterochromatin and euchromatin [117,118]. In particular, *ROS1* specifically targets TEs that are in close proximity to genes and regulates the extent of methylation spreading [119]. Other epigenetic modifications and/or insulator sequences could also restrict TEs’ spreading effects. In mice, the limited spreading of methylation from TEs into nearby genes is associated with the enrichment of active histone modifications combined with the presence of insulator elements at intervening sequences [98]. The same study also postulated that the expression of nearby genes, and, accordingly, the enrichment and “spreading” of active epigenetic marks, may define the boundary of TE-associated enrichment of repressive marks. Consistent with this prediction, it was observed that pericentromeric heterochromatin-mediated silencing is highly sensitive to the promoter strength of the impacted euchromatic genes [120].

Despite these known molecular mechanisms that limit the spreading of repressive marks, TEs’ epigenetic effects are still widely documented in the very same taxa, suggesting hosts cannot entirely prevent the inadvertent effects of epigenetically silencing TEs. To understand why the deleterious spreading effects of TEs exist in the first place, the critical question to ask may instead be “Is the spreading of repressive marks from TEs evolving? If so, how and why?” Although the survey for variation in the epigenetic effects of TEs between genomes is still restricted to a few taxa, these studies unanimously support that the extent of TE-associated enrichment of repressive marks varies within populations [50] and between closely related species [46,57]. These observations indicate that TEs’ epigenetic effects, instead of being static, do evolve. But what evolutionary mechanisms underlie these differences? Similar to the spreading of repressive epigenetic marks from constitutive heterochromatin, the epigenetic effects of TEs depend on the host machinery of epigenetic regulation [121]. Varying epigenetic effects of TEs could thus be driven by variation at the coding sequences and/or regulatory elements of genes that regulate the overall chromatin environment. Consistent with this hypothesis, the 1001 *A*. *thaliana* consortium has identified associations between SNPs in genes involved in the methylation pathway (*CMT2 and AGO9*) and the level of methylation at TEs [50]. There is also a correlation between the expression level of key heterochromatin regulator proteins (*Su(var)*s) and the magnitude of TEs’ epigenetic influence between *Drosophila* species [46].

What evolutionary force might have driven the variation of these host genetic factors in the first place? In other words, why would host genetic factors have evolved to escalate the harmful epigenetic effects of TEs in some species but not the others? Genes involved in epigenetic regulation are highly pleiotropic and are critical players in a wide array of cellular functions [13,122–124]. Accordingly, selection could have acted on their other vital roles. Alternatively, the difference in TEs’ epigenetic effects between species may be the consequence of varying TE abundance, instead of the cause. A significant change in TE abundance in host genomes could also be driven by changes in host effective population sizes [125], host mating systems [126,127], or horizontal transfer of TEs between hosts [128]. This increase in TE copy number could result in selection for weakening the epigenetic silencing of TEs to reduce the extent and thus deleteriousness of the associated spreading effects. However, this reduced suppression of TEs could lead to an increased rate of TEs’ selfish replication and, accordingly, even more TEs in host genomes. On the other hand, in response to an increased abundance of TEs, selection may instead favor stronger epigenetic silencing of TEs to prevent TEs’ selfish increase, although theories predict that selection for reduced TE replication only happens under restricted conditions [129]. In either scenario, the strength of silencing TEs and thus TEs’ epigenetic effects could fluctuate between states without reaching a stable equilibrium, leading to hosts never having perfect control of the harmful spreading effects of TE silencing. Similar ideas were put forward in the “auto-immunity” hypothesis, which was proposed to explain the rapid evolution of proteins in the small-RNA pathway [130]. Specifically, in response to the constant invasion of novel TE families through horizontal transfer, the small-RNA pathway experiences oscillating selection that favors either increased sensitivity to target newly invaded TE families or increased specificity to limit deleterious off-target effects from small-RNA–mediated epigenetic silencing of TEs. In addition to varying host epigenetic environment, differential targeting of TEs by the fast-evolving small-RNA pathway could be another driving force for the differences in TE-mediated spreading effects between taxa.

## Future outlook

A preponderance of studies supports that TEs’ epigenetic effects are prevalent across both animal and plant genomes and can significantly influence host fitness. This spreading effect can extend over substantial distances, potentially impacting more genes and functional elements than some of the genic impacts of TEs. However, many unanswered questions about this previously overlooked effect of TE still await future investigations. Curiously, between species, within genomes, or even among copies of the same TE family, there is considerable variability in the epigenetic effects of TEs. Especially intriguing is how some genomes are infested with TEs but still have quite extensive epigenetic effects of TEs (e.g., maize, approximately 1 kb), while other species of low TE content show a more restricted spreading of repressive marks (e.g., *A*. *thaliana*, much less than 1 kb). TE-mediated spreading of repressive marks generally associates with reduced expression of neighboring genes, with many exceptional cases of this overall trend. Furthermore, the transcriptional influence of TEs’ epigenetic effects is yet to be disentengled from other *cis* effects of TEs. In fact, the relative importance of TEs’ epigenetic and genetic effects in the evolution of both hosts and TE themselves is an important but still largely unexplored question. One of the predicted evolutionary consequences of TEs’ epigenetic effects is the impact on genome evolution through recombination suppression in the vicinity of TEs. Beyond the TE neighborhood, TE-mediated spreading effects could potentially broadly shape the evolution of an entire chromosome or even the whole genome on a long evolutionary time scale, such as the formation of nonrecombining sex chromosome (reviewed in [81]) or the subgenome dominance in allopolyploids (reviewed in [131,132]). When investigating these unanswered questions regarding the causes and consequences of TE-mediated spreading effects, integration of genetic and molecular experiment, phylogenetically informed comparative epigenomic studies (e.g., a multispecies extension of [46]), and theoretical analysis (including simulations, revision of classic TE models, or development of new models (e.g., [133])) will provide important novel insights to further push forward our understanding of TEs’ epigenetic influences. The observed prevalence, fitness impact, and unique evolutionary consequences highlighted by this Review emphasize that TEs’ epigenetic effects are a crucial component not only for TEs’ biology but also for the function and evolution of host genomes.
